# Coordinated Epigenetic Regulation in Plants: A Potent Managerial Tool to Conquer Biotic Stress

**DOI:** 10.3389/fpls.2021.795274

**Published:** 2022-01-03

**Authors:** Chien-Yu Huang, Hailing Jin

**Affiliations:** Department of Microbiology and Plant Pathology, Institute for Integrative Genome Biology, University of California, Riverside, Riverside, CA, United States

**Keywords:** epigenetics, DNA methylation, histone modification, small RNAs, chromatin remodelers, plant defense, plant-microbes interaction

## Abstract

Plants have evolved variable phenotypic plasticity to counteract different pathogens and pests during immobile life. Microbial infection invokes multiple layers of host immune responses, and plant gene expression is swiftly and precisely reprogramed at both the transcriptional level and post-transcriptional level. Recently, the importance of epigenetic regulation in response to biotic stresses has been recognized. Changes in DNA methylation, histone modification, and chromatin structures have been observed after microbial infection. In addition, epigenetic modifications may be preserved as transgenerational memories to allow the progeny to better adapt to similar environments. Epigenetic regulation involves various regulatory components, including non-coding small RNAs, DNA methylation, histone modification, and chromatin remodelers. The crosstalk between these components allows precise fine-tuning of gene expression, giving plants the capability to fight infections and tolerant drastic environmental changes in nature. Fully unraveling epigenetic regulatory mechanisms could aid in the development of more efficient and eco-friendly strategies for crop protection in agricultural systems. In this review, we discuss the recent advances on the roles of epigenetic regulation in plant biotic stress responses.

## Introduction

Epigenetic modification is a regulatory mechanism of gene expression caused by changes in chromatin structure and function without alteration of the DNA sequence. Epigenetic regulation of both mammalian and plant genomes has been intensely studied over the last two decades. Unlike animals, plants are sessile and unable to escape from variable environmental extremes or biotic stressors like herbivores and pathogens. To withstand pathogens and pests, plants have evolved highly sophisticated defense regulatory mechanisms (Jones et al., 2016; Arnold et al., 2019; Bakhtiari et al., 2019; Zhou and Zhang, 2020). A growing number of studies have unveiled that epigenetic regulation is crucial for shaping plant immunity and phenotypic variations during plant-microbe interaction.

Plants lack an adaptive immune system or specialized cells for immune response. Plants detect conserved pathogen-associated molecular patterns (PAMPs) *via* the host plasma membrane-associated pattern recognition receptors (PRRs). This recognition triggers general nonspecific immune responses, named PAMP-triggered immunity (PTI; Bigeard et al., 2015; Nürnberger and Kemmerling, 2018). To suppress PTI and promote a successful invasion, pathogens evolved the ability to send effectors into plant cells to modulate host gene expression and suppress plant immunity (Irieda et al., 2019). In turn, host cells have evolved the ability to recognize pathogen effectors using intracellular nucleotide-binding domain and leucine-rich repeat receptors (NLRs), which activate innate immune responses against pathogen infection (Jones et al., 2016; Zhou and Zhang, 2020). The recognition of effectors by NLRs induces rapid and robust effector-triggered immunity (ETI; Cui et al., 2015; Lolle et al., 2020). Expression and activation of NLRs are precisely regulated at many different levels, including transcriptional regulation, alternative transcript splicing, non-coding small RNA (sRNA)-mediated post-transcriptional regulation, translational regulation, post-translational modification, NLR dimerization or oligomerization, and proteasome-mediated degradation (Li et al., 2015; Lai and Eulgem, 2018). Failure of NLR activation or over accumulation of NLRs can lead to serious diseases or autoimmune responses, respectively. In plants, the local defense response triggers subsequent systemic acquired resistance (SAR) in distal leaves to prevent or reduce future infection (Fu and Dong, 2013). PTI, ETI, and SAR are associated with elevated levels of the phytohormone salicylic acid (SA), a phenolic compound produced by a wide range of prokaryotic and eukaryotic organisms. Besides SA, jasmonic acid (JA) and ethylene are also important phytohormones in biotic stress signaling (Burger and Chory, 2019). In addition, chemicals, such as microbe or host-derived molecules, can act as priming stimuli by inducing disease resistance, which enables a more robust response by the host defense system to future attacks. Further, this ability can sometimes be transmitted to progeny (Conrath et al., 2015; Mauch-Mani et al., 2017; Lopez Sanchez et al., 2021). This transgenerational priming is highly correlated to inherited epigenetic modifications.

Epigenetic regulation of gene expression involves various components, including enzymes that catalyze or remove DNA methylation and histone post-translational modification (PTM), sRNAs, and chromatin remodelers. Together, these components contribute to the precise integrated transcriptional regulation of gene expression. Molecular interactions, including protein-protein, protein-DNA, protein-RNA, and RNA-DNA-protein complexes, establish, erase, or edit epigenetic marks on both genomic DNA or histones to alter chromatin structures and accessibility. The major roles of DNA methylation include the maintenance of plant genome stability by inhibiting the movement of transposable elements (TEs), and the repression of gene expression by recruiting gene expression suppressors or preventing the binding of transcription factors to the methylated DNA. Heterochromatic siRNAs (hc-siRNA) are associated with RNA-directed DNA methylation (RdDM) involved in the deposition of DNA methylation and transcriptional gene silencing (TGS; Kim and Zilberman, 2014; Matzke and Mosher, 2014; Bond and Baulcombe, 2015; Erdmann and Picard, 2020). PTMs at the N-terminal tails of core histones (H2A, H2B, H3, and H4) impact the interaction of histones with DNA, transcription factors, and chromatin remodelers to regulate gene expression (Liu et al., 2010; Deal and Henikoff, 2011). In addition, DNA methylation and histone H1 jointly maintain transcriptional homeostasis by silencing TEs and aberrant intragenic transcripts (Choi et al., 2020). In plants, emerging evidence shows that various epigenetic regulatory mechanisms including DNA methylation dynamics, changes in histone density and variants, and histone PTMs play important roles in regulating plant defense responses (Deleris et al., 2016; Ramirez-Prado et al., 2018; Huang et al., 2019). Different histone marks, together with their specific writers, readers, and erasers coordinate the transcription of defense-related genes (Ramirez-Prado et al., 2018; Hu et al., 2019). While the role of chromatin remodelers and their crosstalk with DNA methylation and histone modification in regulating plant growth and development has been extensively studied (Han et al., 2015), their potential roles in regulating plant immune responses require further investigation. Here, we review recent discoveries on epigenetic regulation of plant immune responses, including the responses of the *Arabidopsis* mutant plants of DNA methylation components, histone modification readers, and chromatin remodelers to non-viral pathogens (Table 1), and in crop diseases (Table 2).

**Table 1:** The responses of the *Arabidopsis* mutants plants of DNA methylation components, histone readers, and chromatin remodelers to different pathogens.

Arabidopsis mutants	Phenotype	DNA methylation	Defense response	References
**RdDM pathway components**				
*drm1/drm2/cmt3* (*ddc*)	Resistant to *Pst*, *Pst*(*AvrPphB*) and *Pst*(*hrc*^−^) (bacterium)	hypo	SA-dependent response is enhanced	Dowen et al., 2012; Luna et al., 2012; Yu et al., 2013; Cambiagno et al., 2021
	Susceptible to *Ab* (necrotrophic fungus)		JA-dependent defense is suppressed	Luna et al., 2012
	Susceptible to *At* (bacterium)		ABA-dependent response is enhanced	Gohlke et al., 2013
*drm1/drm2*	Susceptible to *Pc* (necrotrophic fungus)	hypo	JA-dependent defense is suppressed	Lopez et al., 2011
	Resistant to *Pst*		Primed state of defenses response	Yu et al., 2013; Cambiagno et al., 2021
*nrpd1* (PolIV)	Resistant to *Pst*	hypo	SA-dependent response is enhanced	Dowen et al., 2012
*nrpe1* (PolV)	Resistant to *Pst*	hypo	SA-dependent response is enhanced	Lopez et al., 2011
	Resistant to *Hpa* (biotrophic oomycete)		SA-dependent response is enhanced	Lopez Sanchez et al., 2016
	Susceptible to *Pc* and *Bc* (fungus)		JA-dependent defense is suppressed	Lopez et al., 2011; Lopez Sanchez et al., 2016
*nrpd1/nrpe1* (PolIV/ PolV)	Resistant to *Pst*	hypo	SA-dependent response is enhanced	Lopez et al., 2011
	Susceptible to *Pc*		JA-dependent defense is suppressed	Lopez et al., 2011
*nrpd2* (shared by PolIV and PolV)	Resistant to *Pst*	hypo	SA-dependent response is enhanced	Lopez et al., 2011; Yu et al., 2013
	Susceptible to *Pc* and *Bc*		JA-dependent defense is suppressed	Lopez et al., 2011
*drd1*	Susceptible to *Pc*	hypo	JA-dependent defense is suppressed	Lopez et al., 2011
	Resistant to *Pst*		SA-dependent response is enhanced	Dowen et al., 2012
	Resistant to *Hpa*		SA-dependent response is enhanced	Lopez Sanchez et al., 2016
*ago4*	Susceptible to *Pst*	hypo	RDR2 and DCL3 independent susceptibility	Agorio and Vera, 2007
	Susceptible to *At*		ABA-dependent response is enhanced	Gohlke et al., 2013
*rdr2*	Resistant to *Pst*	hypo	SA-dependent response is enhanced	Dowen et al., 2012
	Susceptible to *Pc*		JA-dependent defense is suppressed	Lopez et al., 2011
*rdr6*	Susceptible to *Pst*(*AvrRpt2*)	–	Loss sRNAs contribute to *RPS2*-mediated *ETI*	Katiyar-Agarwal et al., 2006
	Resistant to *Pst*		–	Dowen et al., 2012
	Susceptible to *Bc*		Loss the transfer siRNAs targets pathogen genes	Cai et al., 2018
*dcl2/3/4*	Resistant to *Pst*	hypo	–	Dowen et al., 2012
	Susceptible to *Bc*	–	Loss the transfer siRNAs targets pathogen genes	Cai et al., 2018
*DNA methylation* *(drm1/drm2/cmt3 (ddc)* and *drm1/drm2 see above)*				
*cmt3*	Resistant to *Hpa*	hypo	SA-dependent response is enhanced	Lopez Sanchez et al., 2016
*met1*	Resistant to *Pst*, *Pst*(*AvrPphB*) and *Pst*(*hrc*^−^)	hypo	SA-dependent response is enhanced	Dowen et al., 2012; Yu et al., 2013
**DNA demethylation**				
*ros1*	Susceptible to *Pst*	hyper	Methylation at the promoter of *RMG1* and *RLP43*	Yu et al., 2013; Halter et al., 2021
	Resistant to *Pc*		JA-dependent defense is enhanced	Lopez Sanchez et al., 2016
*ros1/dml2/dml3* (*rdd*) and *rdd DME* RNAi	Susceptible to *Fo* (hemibiotrophic vascular fungus)	hyper	*Fo* responses gene is suppressed by DNA methylation	Schumann et al., 2019
**Chromatin remodelers and epigenetic regulators**				
*edm2*	Susceptible to *Hpa*	–	Control expression of *RPP7*	Eulgem et al., 2007
	Resistant to *Pst*		NLRs unsuppressed	Lai et al., 2020
*pie1*(*swr1*)	Resistant to *Pst*	–	Constitutive SAR response	March-Diaz et al., 2008
*clsy1*	Progeny is not prime to against *Hpa*	–	Transgenerational SAR is impaired	Luna and Ton, 2012
*ddm1*	Resistant to *Pst*	hypo	SA-dependent response is enhanced	Stokes et al., 2002; Cambiagno et al., 2021
*syd-4*	Resistant to *Pst*	–	*SNC1* expression is enhanced	Johnson et al., 2015
*swp73a*	Resistant to *Pst*(*AvrRpt2*) and *Pst*(*AvrRps4*)	–	NLRs unsuppressed	Huang et al., 2021

*Pathogens include bacterial pathogens Pseudomonas syringae pv. tomato DC3000 (Pst, secreted effectors AvrPphB, AvrRpt2, AvrRps4, and type III secretion system mutant hrc^−^) and Agrobacterium tumefaciens (At), biotrophic oomycete Hyaloperonospora arabidopsidis (Hpa), hemibiotrophic vascular fungus Fusarium oxysporum (Fo), fungus Botrytis cinerea (Bc), Magnaporthe oryzae (Mo), necrotrophic fungus Plectosphaerella cucumerina (Pc), and Alternaria brassicicola (Ab)*.

**Table 2:** Examples of molecular regulators or treatments which cause epigenetic modification and regulate crop disease response.

Epigenetic modification molecules or treatment	Function	Effect	References
**Rice**			
*OsAGO4a*-RNAi	Reduce siRNA accumulation and CHH methylation at the *PigmS* promoter and enhance *PigmS* expression	The mutant plant is susceptible to *Magnaporthe oryzae* (fungus)	Deng et al., 2017
*TE derived hc-siRNAs*	Control *PigmS* expression	Avoid fitness cost due to the defense response induced by PigmR against *Magnaporthe oryzae*	Deng et al., 2017
*TE derived hc-siRNAs*, *TE-siR815*	Suppress *ST1* expression	Attenuation of *WRKY45*-mediated resistance to *Xanthomonas oryzae* pv. Oryzae (bacteria)	Zhang et al., 2016
miR812w	Targets *Stowaway* MITE to suppress nearby gene	Contribute to *Magnaporthe oryzae* resistance	Campo et al., 2021
**Common bean**			
BABA	H3K4me3 and H3K36me3 are enhanced at the promoter-exon regions of defense-associated genes	Induces resistance to *P. syringae* pv. *phaseolicola*	Martinez-Aguilar et al., 2016
**Potato**			
BABA	Adjust H3K4me2 and H3K27me3 dynamics; and genome-wide DNA hypermethylation	Induces intergenerational resistance against *Phytophthora infestans* (oomycete)	Meller et al., 2018
	Reduce DNA methylation on the promoter of *R3a* NLR gene	More resistant to virulent *Phytophthora infestans* which secretes effector Avr3a	Kuznicki et al., 2019

## Small Rna-Mediated Epigenetic Modification Regulates Plant Defense

In plants, sRNAs, including microRNAs (miRNAs) and small-interfering RNAs (siRNAs), are generated by the type III ribonuclease Dicer or Dicer-like (DCL) proteins and are incorporated into Argonaute (AGO) proteins to induce gene silencing in a sequence-specific manner (Baulcombe, 2004). An sRNA is loaded into an AGO protein and then induces TGS or post-transcriptional gene silencing (PTGS) of their target genes endogenously or even in interacting organisms (Baulcombe, 2004; Weiberg et al., 2013; Cai et al., 2018; Huang et al., 2019). In general, miRNAs are processed from single-stranded primary RNA precursors with stem-loop structures, whereas siRNAs are generated from double-stranded RNAs (dsRNAs) that are derived from invert repeats, sense-antisense transcript pairs, or products of RNA-dependent RNA polymerases (RDRs). Plant siRNAs can be further divided into trans-acting siRNAs (ta-siRNAs; Allen et al., 2005) or secondary phased siRNAs (phasiRNAs; Fei et al., 2013), hc-siRNAs, natural antisense transcripts-derived siRNAs (nat-siRNAs; Katiyar-Agarwal et al., 2006), and long siRNAs (lsiRNAs; Katiyar-Agarwal et al., 2007) based on their biogenesis pathways (Borges and Martienssen, 2015; Huang et al., 2019). Different types of sRNAs were reported to precisely regulate the expression of NLRs and plant defense signaling genes to activate plant immune responses and to balance the trade-off between plant growth and defense (Huang et al., 2019). Here, we mainly focus on the functions of some sRNAs that play a direct role in epigenetic regulation in the following sections.

**Figure 1:**
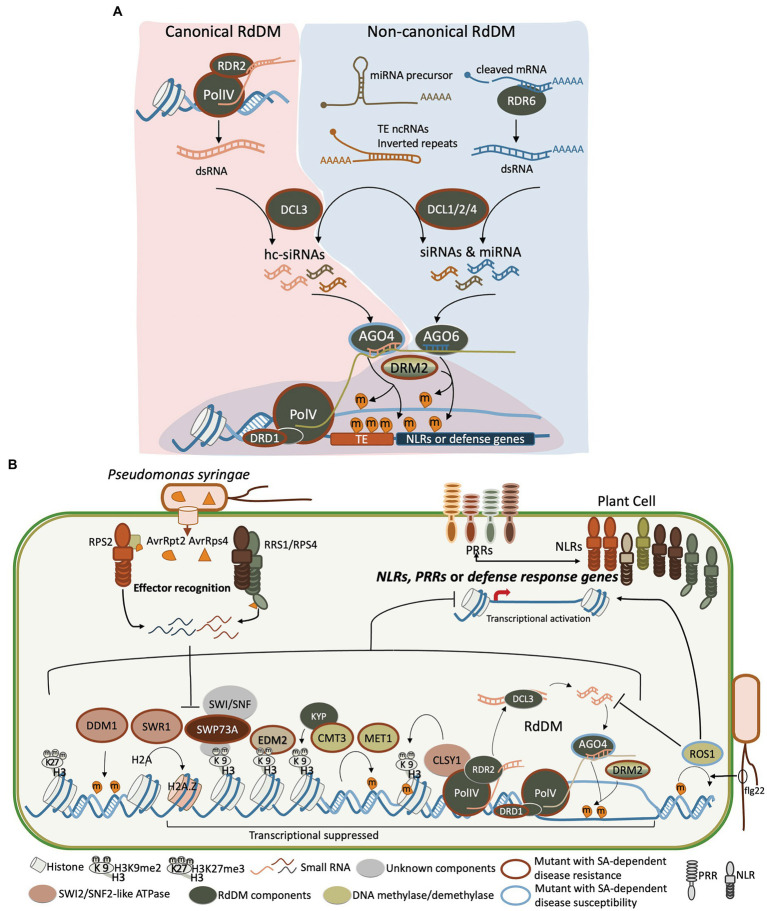
The epigenetic regulatory mechanisms act coordinately in reprogramming gene expression when plants encounter biotic stress. The known factors in *Arabidopsis* that regulate the biotic stress involve small RNAs, RdDM, methylation and demethylation of DNA, histone modification, and chromatin remodelers, which are present here. **(A)** Small RNAs participate in conical and non-canonical RdDM pathways to regulate DNA methylation on TEs and defense-related genes. The RDR2- and DCL3-dependent hc-siRNAs are key components in PolIV-RDR2-DCL3-AGO4-PolV-DRM2, the conical RdDM pathway, which establish and reinforce DNA methylation at TEs and regulate the nearby defense response genes. The siRNAs derived from mRNA precursor, TE non-coding RNAs (TE ncRNAs), inverted repeats, or dsRNAs produced by RDR6 are processed by DCL2/4 also participate in establishing the DNA methylation and regulate the expression of defense-related genes. **(B)** Deposition of DNA methylation and on the NLRs, PRRs, or defense response genes leads to a transcriptionally suppressed status. Other components acting with the chromatin remodeler SWP73A (dark red oval) are not clear (unknown component is shown in gray oval). RdDM components including *PolIV* and *PolV*, *DCLs*, *RDRs*, *AGO4/6*, and *DRD1 are shown in a dark green oval*. DNA methyltransferases *DRM2*, *MET1*, and *CMT3* are shown in a light green oval. SWI2/SNF2-like ATPases *Decreased DNA Methylation 1* (*DDM1*), *Swi2/Snf2-related 1* (*SWR1*), and *Classy1* (*CLSY1*) are shown in an orange oval. The H3K9me2 reader EDM2 is shown in the light brown oval. Mutant plants that display a resistant phenotype to pathogen (such as *Pst*) relying on an SA-dependent response are surrounded with a red outline, whereas the blue outline indicates a more susceptible phenotype.

Heterochromatic siRNAs, which are typically 24–30 nt in length, play a central role in the canonical RdDM pathway (Figure 1A). The biogenesis of hc-siRNAs is dependent on the RDR2-DCL3-AGO4/6/9 pathway and also requires plant-specific RNA polymerase IV and V (PolIV and PolV; Figure 1A). They are derived from TEs, repeats, and heterochromatic regions and act to direct *de novo* DNA methylation and/or histone modifications at the target region (Matzke and Mosher, 2014). Many PRR/NLR loci or clusters are associated with TEs or repeats (Cambiagno et al., 2018) and, thus, are regulated by hc-siRNA-mediated epigenetic regulation. One example of this is the rice NLR *Pigm* locus, which confers durable resistance to the rice blast fungus *Magnaporthe oryzae* (Deng et al., 2017; Table 2). The *Pigm* locus encodes a cluster of NLRs and NLR pseudogenes, including *PigmR* and *PigmS*. Constitutive expression of *PigmR* confers resistance to *M. oryzae* but causes yield losses. *PigmS*, which is highly expressed in pollen and panicles, forms a heterodimer with *PigmR* to suppress *PigmR*-mediated resistance to avoid fitness costs. The *PigmS* promoter contains two tandem miniature transposons (MITEs), which associate with hc-siRNAs. Thus, the precise control of *PigmS* expression is mediated by hc-siRNA at the epigenetic level.

In addition to NLRs, hc-siRNAs also regulate signaling components of plant defense. For example, a rice hc-siRNA, TE-siR815, derived from a MITE in the first intron of the transcription factor *WRKY45-1* allele, induces TGS of a leucine-rich repeat receptor kinase-type protein gene, *ST1*. *ST1* is a key component in the *WRKY45* signaling pathway and this suppression leads to attenuation of *WRKY45*-mediated resistance to bacterial blight of rice caused by *Xanthomonas oryzae* pv. Oryzae (*Xoo*; Zhang et al., 2016; Table 2). Unlike the *WRKY45-1* allele, the *WRKY45-2* allele does not contain the TE-siR815-generating MITE, which allows signaling pathway activation against *Xoo*. Other than hc-siRNA, miRNA can participate in non-canonical RdDM (Cuerda-Gil and Slotkin, 2016). A rice-specific miR812w, which originates from the *Stowaway* MITE, targets *Stowaway* MITE to suppress the expression of nearby genes through miRNA-directed DNA methylation, contributing to *M. oryzae* resistance (Campo et al., 2021; Table 2). Taken together, these studies demonstrate that sRNAs can regulate plant defense response through RdDM.

## Dynamics of Dna Methylation in Response To Biotic Stresses

In plants, DNA methylation is observed on cytosine in the context of symmetric CG and CHG, and asymmetric CHH (where H = A, C, or T). CHH methylation is primarily established by *de novo* DNA methylation through RdDM by the RNA scaffolds produced by PolV, which recruit DNA (cytosine-5)-methyltransferase DRM2 and hc-siRNAs that are produced by RDR2 and DCL3 and associated with AGO4/6/9 in *Arabidopsis*. The PolIV-RDR2-DCL3-AGO4-PolV-DRM2 pathway forms a feedback loop to reinforce DNA methylation at heterochromatin regions and TEs (Figure 1). In addition to this canonical RdDM, DNA methylation can also be established by the RDR6-DCL2-derived 21 and 22 nt siRNA pathway, which is also dependent on AGO4 and AGO6 (Nuthikattu et al., 2013; Cuerda-Gil and Slotkin, 2016). Afterward, spreading of CG and CHG methylation is maintained by DNA (cytosine-5) Methyltransferase 1 (MET1) and a plant-specific Chromomethylase 3 (CMT3), respectively (Zhang et al., 2018). DNA methylation is reversible and the process of DNA demethylation can be passive or active. The passive process occurs in the absence of DNA methylation on newly synthesized DNA strands. Active DNA demethylation requires the direct removal of a methyl group from DNA by DNA demethylases. *Arabidopsis* has four DNA demethylases, including DEMETER (DME), DME-Like 1/Repressor of Silencing 1 (DML1/ROS1), DML2, and DML3 (Kumar et al., 2018).

DNA methylation dynamics are dependent on the equilibrium between methylation and demethylation pathways. In genome-wide regulation, the DNA methylation within repetitive sequences or TEs are altered in response to infection of the bacterial pathogen *Pseudomonas syringae* pv. tomato DC3000 (*Pst*) and SA treatment and subsequently regulate the transcription of neighboring genes (Agorio and Vera, 2007; Dowen et al., 2012). In general, *Arabidopsis* mutant plants with DNA hypomethylation are more resistant to disease and exhibit an elevated SA-dependent response. For example, *met1*, *drm1/drm2* and *drm1/drm2/cmt3* (*ddc*), *nrpd1* (PolIV mutant), *nrpe1* (PolV mutant), *nrpd1/nrpe1*, *nrpd2* (subunit shared by PolIV and PolV), *drd1* (*defective in RNA-directed DNA methylation*), *rdr2*, and *dcl2/3/4* are more resistant to the bacterial pathogen *Pst*; *cmt3*, *drd1*, and *nrpe1* are more resistant to the obligate biotrophic oomycete pathogen *Hyaloperonospora arabidopsidis* (*Hpa*; Lopez et al., 2011; Dowen et al., 2012; Yu et al., 2013; Lopez Sanchez et al., 2016; Cambiagno et al., 2021; Table 1 and Figure 1A).

Conversely, some mutations in sRNA biogenesis and RdDM-dependent pathways are more susceptible to pathogen infection. For example, mutation in *RDR6*, which is required for phasing siRNA and nat-siRNA biogenesis and also mediates non-canonical RdDM, does not have an obvious change on global DNA methylation and is more susceptible to *Pst* strain that secrets effector AvrRpt2, *Pst* (*AvrRpt2*), but displays minor changes in response to *Pst* infection (Katiyar-Agarwal et al., 2006; Dowen et al., 2012; Nuthikattu et al., 2013). AvrRpt2 triggers NLR RPS2-mediated ETI so this suggested that RDR6-mediated siRNAs play a crucial role for RPS2-mediated ETI. The *rdr6* and *dcl2/3/4* mutants, which greatly reduce the biogenesis of siRNAs, are more susceptible to fungal pathogen *Botrytis cinerea* (*Bc*). This susceptible phenotype is caused by the fact that the host plant has lost the siRNAs that move into fungal cells to suppress fungal virulence-related genes (Cai et al., 2018). *Drd1*, *nrpe1*, *nrpd1/nrpe1*, and *nrpd2* also display enhanced susceptibility to the necrotrophic fungus *Plectosphaerella cucumerina* (Lopez Sanchez et al., 2016) and *the ddc* mutant is more susceptible to necrotrophic fungus *Alternaria brassicicola* (Lopez et al., 2011; Luna et al., 2012). This is due to the fact that the defensive signaling against *P. cucumerina* and *A. brassicicola* is JA-dependent, which is suppressed in nrpe1 and ddc mutants. RdDM mutant *ago4* is more susceptible to *Pst* but this phenotype is independent of other upstream components of the RdDM pathway including *RDR2* and *DCL3* (Agorio and Vera, 2007). Thus, this response could be caused by other regulatory mechanism of *AGO4*, which is suggested from the following study. The *ddc* and *ago4* mutants were found to be more susceptible to *Agrobacterium tumefaciens*, the bacterium that causes crown gall tumors. This enhanced susceptibility phenotype was abscisic acid (ABA) dependent, which is also regulated by DNA methylation (Gohlke et al., 2013). ABA plays a pivotal role in abiotic stress responses and has negative impacts on plant immunity against diverse pathogens (Robert-Seilaniantz et al., 2011). This impact is attributed to ABA-mediated suppression of plant immune responses induced by immune hormones SA, JA, and ethylene (Mine et al., 2017). Thus, dynamic changes in DNA methylation in response to pathogen infection play a pivotal role in plant immune responses.

Active demethylation also shapes transcriptional reprogramming of immune response genes upon infection of different pathogens (Table 1). In *Arabidopsis*, the bacterial PAMP, flagellin-derived peptide flg22, derepresses RdDM targeted genes, such as an NLR gene, *RMG1*, and a PRR gene, *RLP43*, through *ROS1*-directed demethylation on their promoters (Yu et al., 2013; Halter et al., 2021; Figure 1B). In addition, ROS1 antagonizes RdDM-dependent methylation at *RMG1* locus, which may also contribute to anti-bacterial response (Halter et al., 2021). The *ros1* mutant shows hypermethylation and is more susceptible to *Hpa*. Conversely, *ros1* is more resistant to *P. cucumerina*, which is associated with JA-dependent defense pathways (Lopez Sanchez et al., 2016). Furthermore, the triple mutant of DNA demethylases *ros1/dml2/dml3* (*rdd*) and the quadruple mutant *rdd DME* RNAi lines display enhanced susceptibility to a hemibiotrophic vascular fungal pathogen, *Fusarium oxysporum*, that causes disease in many important crops. The tissue-specific expression of four DNA demethylases *DME*, *ROS1*, *DML2*, and *DML3* act cooperatively to construct resistance against *F. oxysporum* (Schumann et al., 2019). It was also found that some *Arabidopsis NLR*s can be demethylated by ROS1, DML2, and DML3 within their promoters and transcribed regions (Kong et al., 2020). Thus, active demethylation processed by DNA demethylases regulates defense response genes upon pathogen infection (Table 1).

## Histone Modification and Crosstalk with Dna Methylation Modulate Plant Defense Responses

Post-translational modifications on histone proteins have direct impacts on the chromatin structure and contribute to the transcriptional regulation of gene expression. Histone modification is a reversible process and is modulated by specific writers that add the modification, erasers that remove the modification, or readers that sense the modification and transduce the downstream signaling pathways. Some histone PTMs are associated with specific transcriptional states. In general, H3K4me (methylation of Histone 3 at Lys4), H3K36me, H3K9ac (acetylation of H3 at Lys9), and H3K27ac are markers for transcriptional activation, whereas H3K27me3 is mainly linked to transcriptional silencing of genes (Xiao et al., 2016). H3K9me2 and H3K9me3 are enriched in heterochromatic regions with a high density of TEs or repeats, where they have a constitutive repressive function (Liu et al., 2010; He et al., 2011). In euchromatic regions, H3K9me2 has been observed to span the entire gene and is correlated with low expression levels (Zhou et al., 2010; West et al., 2014).

The epigenetic regulation of defense-related genes mediated by histone modification was comprehensively discussed in recent reviews (Ramirez-Prado et al., 2018; Hu et al., 2019). Here, we emphasize the crosstalk between histone modification and DNA methylation on plant biotic stress as methylation of DNA and H3K9 is highly correlated with gene silencing in eukaryotes. This link between DNA methylation and H3K9 modification was revealed by binding of CMT3 with the histone methyltransferase Kryptonite/SUVH4 (KYP). The interaction of CMT3 and KYP/SUVH4 constitutes a self-reinforcing loop between histone and DNA methylation in plants which is important for TE silencing (Du et al., 2015; Figure 1B). In addition to DNA methylation on TEs regulating the transcription of neighboring NLR genes (Dowen et al., 2012), some NLR loci or clusters associated with TEs are also controlled by histone marks, such as H3K9me2 (Lai and Eulgem, 2018). Through *Arabidopsis* mutant screening, the methylation of DNA and H3K9 was observed to regulate resistance against *Pst* infection (Cambiagno et al., 2021) and infestation by the pest, green peach aphid *Myzus persicae* (Annacondia et al., 2021). The study revealed that *nrpd1* and *kyp* mutant plants are more resistant to aphids (Annacondia et al., 2021). Whether and how does the crosstalk between DNA and H3K9 methylation contribute to aphid resistance is still largely unknown. The *drm1/drm2*, *ddc*, and *suvh4/5/6* mutant plants do not exhibit constitutive expression of the defense gene marker *PR1* (PATHOGENESIS-RELATED GENE 1) but are more resistant to *Pst* due to a faster and stronger *PR1* induction after *Pst* infection compared to wild-type plants (Cambiagno et al., 2021). This suggest that *drm1/drm2*, *ddc*, and *suvh4/5/6* mutant plants acquired a primed state of defense against *Pst*, which is regulated by both DNA and H3K9 methylation levels. Another example of genes participating in the crosstalk of DNA and H3K9 methylation is *Increase in Bonsai Methylation 1* (*IBM1*), which encodes a histone demethylase and directly associates with the gene body that has the repressive mark, H3K9me2. IBM1 removes mono- and dimethylation of histone lysines and negatively regulates DNA methylation at CHG loci in the genic regions. *IBM1* positively regulates *Arabidopsis* defense responses against *Pst* at the chromatic level by derepressing the defense marker genes *PR1*, *PR2*, and the PTI marker *FRK1* (Chan and Zimmerli, 2019). Thus, the crosstalk between histone modification and DNA methylation contributes to the epigenetic regulation of gene expression in response to pathogen infection.

## Chromatin Remodelers and Epigenetic Regulators Modulate Plant Immunity

While defense-related genes are regulated by covalent DNA and histone modifications, chromatin remodeling proteins also play an important role in regulating NLRs, plant defense signaling components, SA-, and JA-pathway genes. Conserved chromatin remodeling complexes are composed of multiple subunits which regulate gene expression by altering nucleosome composition and interactions at the chromatin structure level. The SWI/SNF chromatin remodeling complexes were initially identified from *Saccharomyces cerevisiae*. They have been broadly studied in many different organisms and can either “read” or “shape” the chromatin landscapes to regulate gene transcription (Raab et al., 2015; Pulice and Kadoch, 2016). SWI/SNF complexes facilitate the activation or repression of the target gene transcription by binding to the DNA or interacting with histones and transcription factors (Zhu et al., 2013; Grossi et al., 2020).

*In Arabidopsis*, the SWI/SNF complex has a well-established role in gene expression regulation in plant growth and development (Han et al., 2015), but only a few studies link the function of SWI/SNF complex subunits to plant immunity (Figure 1B). Most studies on the role of SWI/SNF complexes in plant defense response focus on the SWI2/SNF2-like ATPase subunits. For instance, Swi2/Snf2-related 1 (SWR1) complex replaces the histone H2A with the histone variant H2A.Z to maintain the suppression of several SA-dependent defense genes (March-Diaz et al., 2008). These genes include CLASSY1 (CLSY1), which is implicated in the RdDM pathway, is required for accumulation of hc-siRNA, and interacts with H3K9 methylation (Luna and Ton, 2012; Zhang et al., 2013; Zhou et al., 2018), as well as DDM1, which is required for DNA methylation and regulates expression of NLRs (Jeddeloh et al., 1998, 1999; Stokes et al., 2002; Li et al., 2010; Cambiagno et al., 2021). In addition, the expression of NLR *SNC1* is suppressed by SPLAYED (SYD), another SWI2/SNF2-like ATPase, which is confirmed by the elevated transcription in the *syd* mutant. However, no direct SYD binding site or DNA region has been identified (Walley et al., 2008; Johnson et al., 2015). This could be a result of indirect regulation mediated by SYD.

Other than the SWI2/SNF2-like ATPase subunits, a recent study revealed that *Arabidopsis SWP73A*, a SWI/SNF2 non-ATPase subunit and an ortholog of the mammalian BRG1-Associated Factor 60 (*BAF60*), acts as a negative regulator of a group of NLRs to prevent autoimmunity in the absence of pathogens (Huang et al., 2021). Upon infection of *Pst* (AvrRpt2) or *Pst* (AvrRPS4), *SWP73A* is silenced by two bacterial-induced sRNAs post-transcriptionally, which allows rapid induction of these NLRs to activate plant immune responses (Figure 1B). For some NLRs, such as *RPS2* and *ZAR1*, SWP73A binds with H3K9me2 at their transcription starting site and promoter regions directly to potentiate its suppression function on the expression of these NLRs. For some other NLRs, such as *RPS4* and *RRS1*, SWP73 does not bind to their promoters and transcription starting sites, but instead suppresses their expression indirectly by suppressing *Cell Division Cycle 5* (*CDC5*), a key regulator of RNA splicing, which subsequently interferes with the alternative splicing of these NLRs (Huang et al., 2021). This finding uncovers a new layer of epigenetic control over the precise regulation of NLRs. Potential roles of other SWI/SNF complex subunits in plant immunity remain to be explored.

Other epigenetic regulators, such as *ENHANCED DOWNY MILDEW 2* (*EDM2*), also help modulate the expression of *Arabidopsis* NLRs (Eulgem et al., 2007; Lai et al., 2020). EDM2 binds to the H3K9me2 at the proximal polyadenylation sites of *RPP7* and *RPP4*, which suppresses the maturation of the short transcripts and promotes the accumulation of full length functional *RPP7* and *RPP4* (Tsuchiya and Eulgem, 2013). *EDM2* binds to H3K9me2 at TEs inside or near NLR genes and plays a role in balancing transcript levels of these NLRs. While the expression level of full length *RPP7* mRNA increases, EDM2 also represses the expression of other NLRs, which is evidenced by the fact that the *edm2* mutant is more resistant to *Pst*.

## Epigenetic Memories and Defense Priming

Biotic stress-induced epigenetic changes, triggered by bacteria, fungi, or insect herbivory, can sometimes be transmitted to the progeny, leading to transgenerational priming (Luna et al., 2012; Rasmann et al., 2012; Lopez Sanchez et al., 2021; Moran-Diez et al., 2021). A global clustering DNA methylation study revealed transgenerational acquired resistance-related patterns, which were identified after three generations of *Pst* exposure. The major change to DNA methylated regions occurred at the CG context in gene bodies (Stassen et al., 2018). A screening for *Hpa*-resistant *Arabidopsis* was performed using epigenetic recombinant inbred lines (epiRILs) generated from *ddm1* mutant, which has reduced DNA methylation in all sequence contexts, crossing to wild-type plants. The selected *Hpa*-resistant lines had no growth defect and a stronger *PR1* induction after *Hpa* infection compared to wild-type plants which revealed a priming of SA-inducible defenses. Through transcriptome and DNA methylome analysis of these *Hpa*-resistant epiRILs lines, it was found that genome-wide priming of defense-related genes is sufficient to provide quantitative disease resistance and is heritable (Furci et al., 2019). Therefore, *Arabidopsis* epigenomic responses at the DNA methylation level in previous generations could contribute to transgenerational acquired resistance.

In addition, several studies have revealed that chemical treatment can lead to epigenetic adjustment for enhanced plant disease resistance. For instance, β-aminobutyric acid (BABA) treatment primes *Arabidopsis* PTI against the necrotrophic bacteria, *Pectobacterium carotovorum*. This priming is mediated by H3K9K14ac and H3K4me2 (Po-Wen et al., 2013). Additionally, treatment with BABA or 2,6-dichloroisonicotinic acid in the common bean leads to enhancement in H3K4me3 and H3K36me3 at the promoter-exon regions of defense-associated genes (Martinez-Aguilar et al., 2016; Table 2). BABA treatment also has priming effects in potatoes and induces intergenerational resistance against oomycete *Phytophthora infestans* through epigenetic adjustment of H3K4me2 and H3K27me3 dynamics. After BABA treatment, H3K4me2 was shown to be transiently induced in *NPR1* (*Non-expressor of PR genes*) and *SNI1* (*Suppressor of NPR1 Inducible 1*) resulting in tuning of the SA-responsive gene and enhanced occupancy on the gene body of defense response genes *WRKY1*, *PR1*, and *PR2* in primed plants and their descendants (Meller et al., 2018; Table 2). Progeny of the BABA-primed potato was shown to carry lower DNA methylation on the promoter of *R3a* NLR gene with a higher transcription level of *R3a* and activate to virulent *P. infestans* which secretes effector Avr3a (Kuznicki et al., 2019; Table 2). Thus, the priming response from chemical-primed treatment is highly regulated at the DNA and histone methylation levels.

## Conclusion and Perspectives

Here, we reviewed recent advances on the regulatory role of epigenetic mechanisms, including RdDM, DNA methylation dynamics, histone modifications, and chromatin remodeling, in plant immune responses. The role of hc-siRNAs in guiding DNA methylation endogenously in the RdDM pathway has been well demonstrated, but whether hc-siRNAs can also direct cross-kingdom DNA methylation of target genes in interacting pathogens and organisms remains to be investigated. Epigenetic modifications, including DNA methylation and histone modification, could be heritable without the need to introduce an initial trigger for targeted manipulation, making it an attractive approach to modify a locus for the desired disease-resistant trait. Notably, the components needed to manipulate methylation can be delivered by direct application of RNAs, protein regulators, or priming molecules to plant cells (Mauch-Mani et al., 2017; Que et al., 2019; Gallego-Bartolome, 2020; Watanabe et al., 2021). Therefore, it is possible to bypass genetic transformation to manipulate gene expression through epigenetic modification in economically important crops.

A major challenge in crop management lies in the multiple biotic and abiotic stresses occurring concurrently in the field. The limited information of crosstalk between abiotic stress and biotic stress responses in crop plants makes it difficult to develop strategies to trigger an efficient broad-spectrum resistance response. Though studies probing the relationship between epigenetic regulation and plant biotic stress are emerging, the connection between epigenetic modification at gene loci and disease-resistant traits in different varieties of crops still needs further exploration. In the future, this could become even more important to deal with unpredictable effects due to climate change.

## Author Contributions

C-YH and HJ wrote the manuscript and organized the table and figure. All authors contributed to the article and approved the submitted version.

## Funding

We appreciate the support for Dr. Hailing Jin’s laboratory from the National Institute of Health (R35 GM136379), the National Science Foundation (IOS2017314), the United States Department of Agriculture National Institute of Food and Agriculture (2021-67013-34258 and 2019-70016-29067), the Australian Research Council Industrial Transformation Research Hub (IH190100022), and the CIFAR Fungal Kingdom fellowship to HJ.

## Conflict of Interest

The authors declare that the research was conducted in the absence of any commercial or financial relationships that could be construed as a potential conflict of interest.

## Publisher’s Note

All claims expressed in this article are solely those of the authors and do not necessarily represent those of their affiliated organizations, or those of the publisher, the editors and the reviewers. Any product that may be evaluated in this article, or claim that may be made by its manufacturer, is not guaranteed or endorsed by the publisher.
